# Supplementary material for: Blood Eosinophils Are Associated with Efficacy of Targeted Therapy in Patients with Advanced Melanoma
Source: Cancers (Basel). 2022 May 4;14(9):2294. doi: 10.3390/cancers14092294 (PMC9104271; doi:10.3390/cancers14092294)
Supplement: Supplementary file 1 [file cancers-14-02294-s001.zip › cancers-1684558-supplementary.pdf]

cancers

MDPI

Supplementary Materials

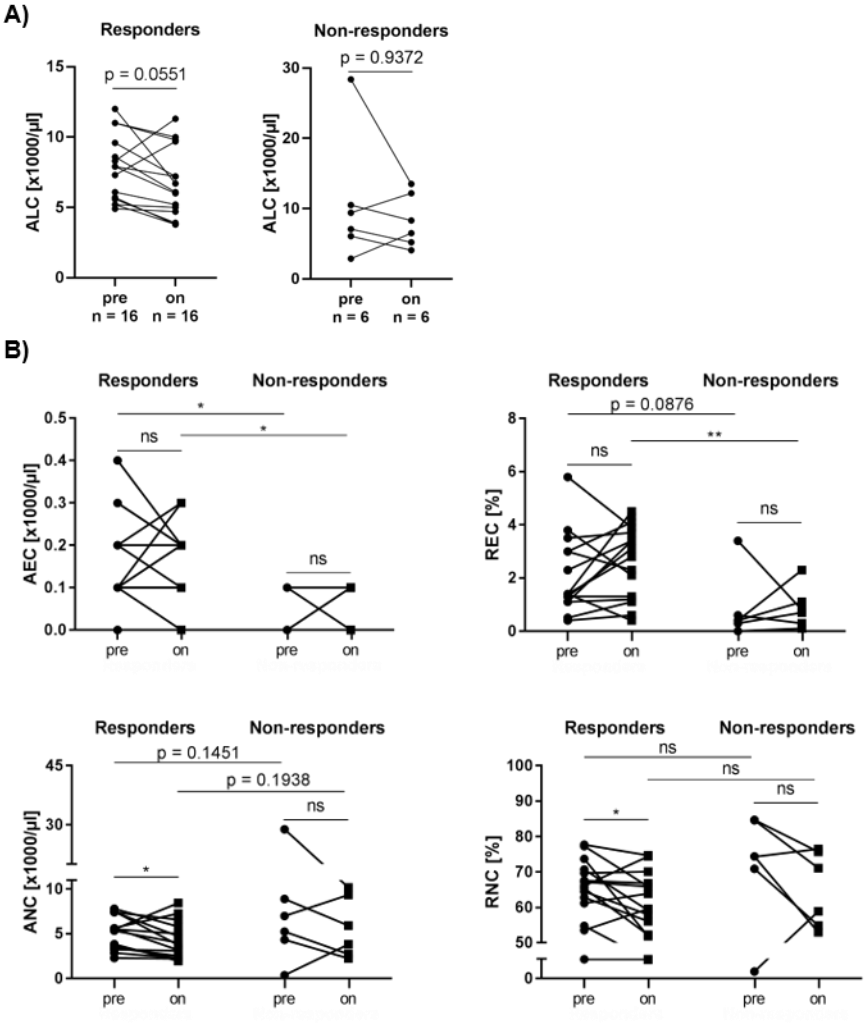

Figure S1. Analysis of leukocyte counts in melanoma patients upon targeted therapy. Samples from 16 responders and six non-responders prior (pre) and during (on) treatment. Results are shown as (**A**) absolute leukocyte count (ALC) and (**B**) absolute and relative eosinophil (AEC, REC) and neutrophil count (ANC, RNC). Responders are characterized as CR and PR, non-responders as PD. ns $p > 0.05$, * $p < 0.05$, ** $p < 0.01$.

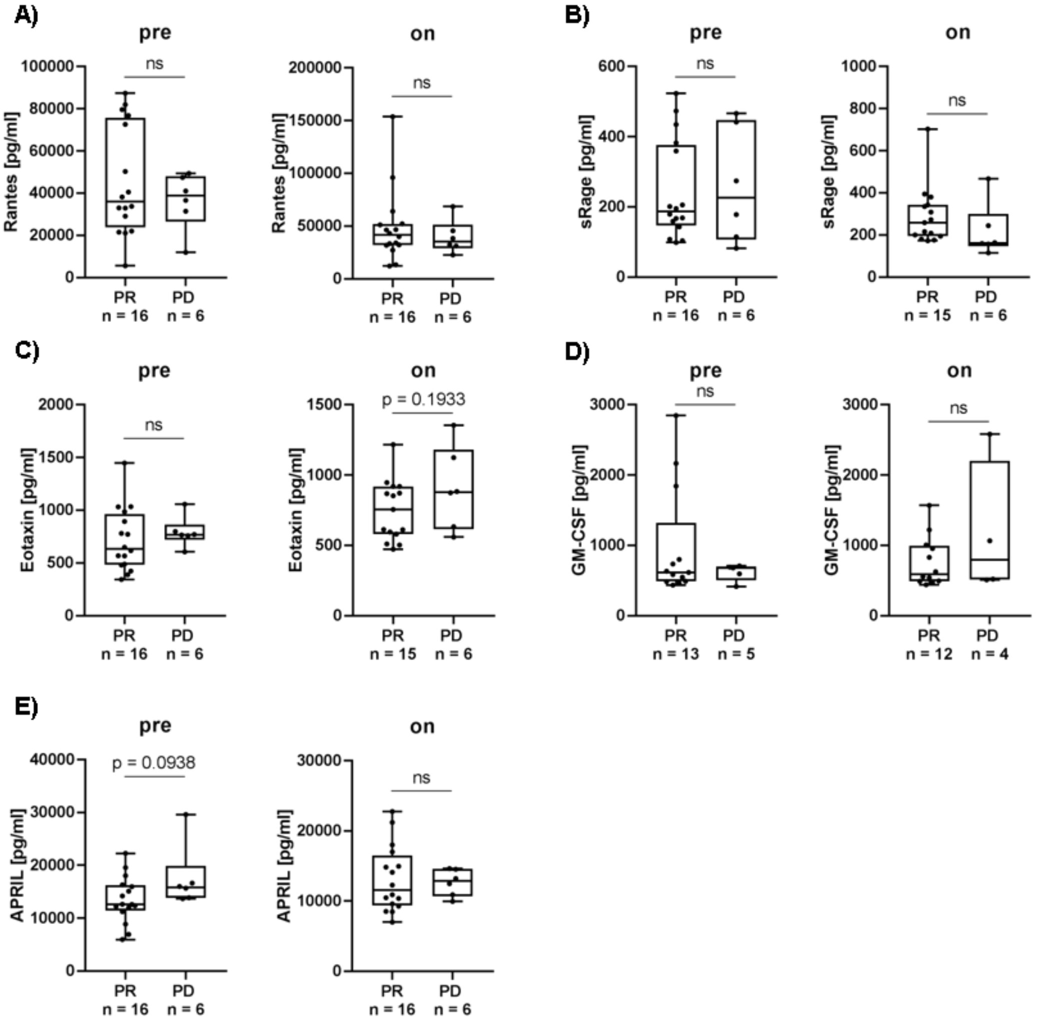

Figure S2. Assessment of eosinophil-related soluble factors in melanoma patient serum prior (pre) and during (on) targeted therapy. Responders are characterized as PR, non-responders as PD. Analysis of (**A**) Rantes (=CCL5), (**B**) sRAGE, (**C**) Eotaxin, (**D**) GM-CSF and (**E**) APRIL before and during drug administration. ns $p > 0.05$.

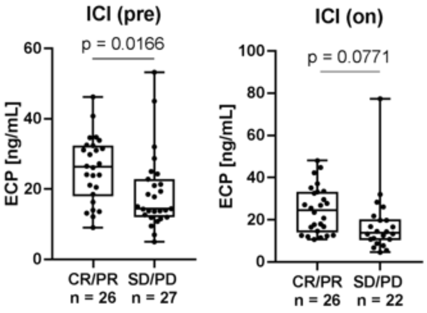

Figure S3. Association of serum ECP with response to immunotherapy. Comparison of serum ECP concentration (ng/mL) of responders and non-responders (**left**) prior (ICI pre) and (**right**) during (ICI on) immunotherapy. Responders to ICI show significant higher pre-treatment serum ECP concentration (p = 0.01) compared to non-responders. This is also numerically (p = 0.07) seen during drug administration.

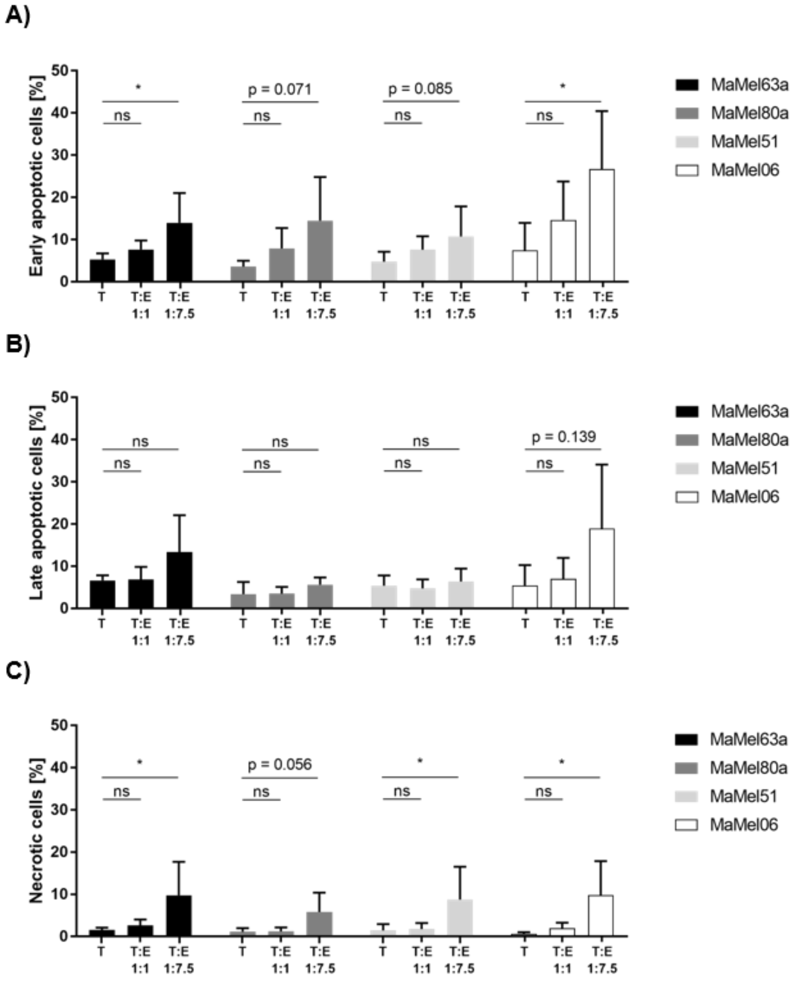

Figure S4. Eosinophils induce apoptosis in various melanoma cell lines. Non-adherent (co-)cultures of melanoma cell lines (T) with or without eosinophils (E) in a ratio of 1:1 and 1:7.5 for 24 hours. (**A**) Mean percentage of melanoma cells in early apoptosis (Annexin V-positive cells), (**B**) in late apoptosis (7-AAD- and Annexin V-positive cells) and (**C**) mean percentage of necrotic melanoma cells (7-AAD-positive cells) ± standard deviation (SD) upon culture or exposure to eosinophils are shown for five to seven independent experiments. ns p > 0.05, * p < 0.05.

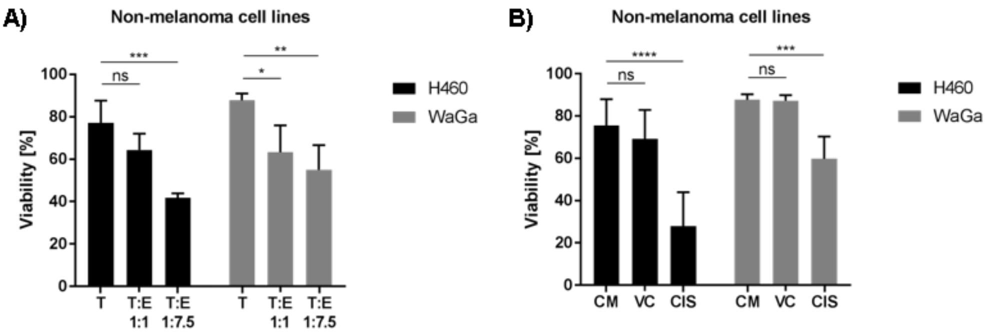

Figure S5. Eosinophils exert tumoricidal activity towards non-melanoma cancer entities to different extents. (**A**) Cytotoxicity assessed in the lung carcinoma cell line, H460 and in the merkel-cell carcinoma cell line, WaGa. Non-adherent (co-)cultures of H460 or WaGa cells with or without eosinophils in a ratio of 1:1 and 1:7.5 cancer cell to effector cell ratio for 24 hours. Eosinophils significantly decrease H460 and WaGa cell viability. A 1:1 ratio is sufficient to significantly impair WaGa cell viability. (**B**) Non-adherent cultures of H460 or WaGa cells with or without combinatory treatment with 1 μM vemurafenib and 100 nM cobimetinib or with 20 μM Cisplatin for 24 hours. Both cell lines are unaffected by targeted therapy but Cisplatin notably reduces cell viability. Mean percentage of the tumor viability ± standard deviation (SD) are shown for four to eight independent experiments. ns $p > 0.05$, * $p < 0.05$, ** $p < 0.01$, *** $p < 0.001$, **** $p < 0.0001$.

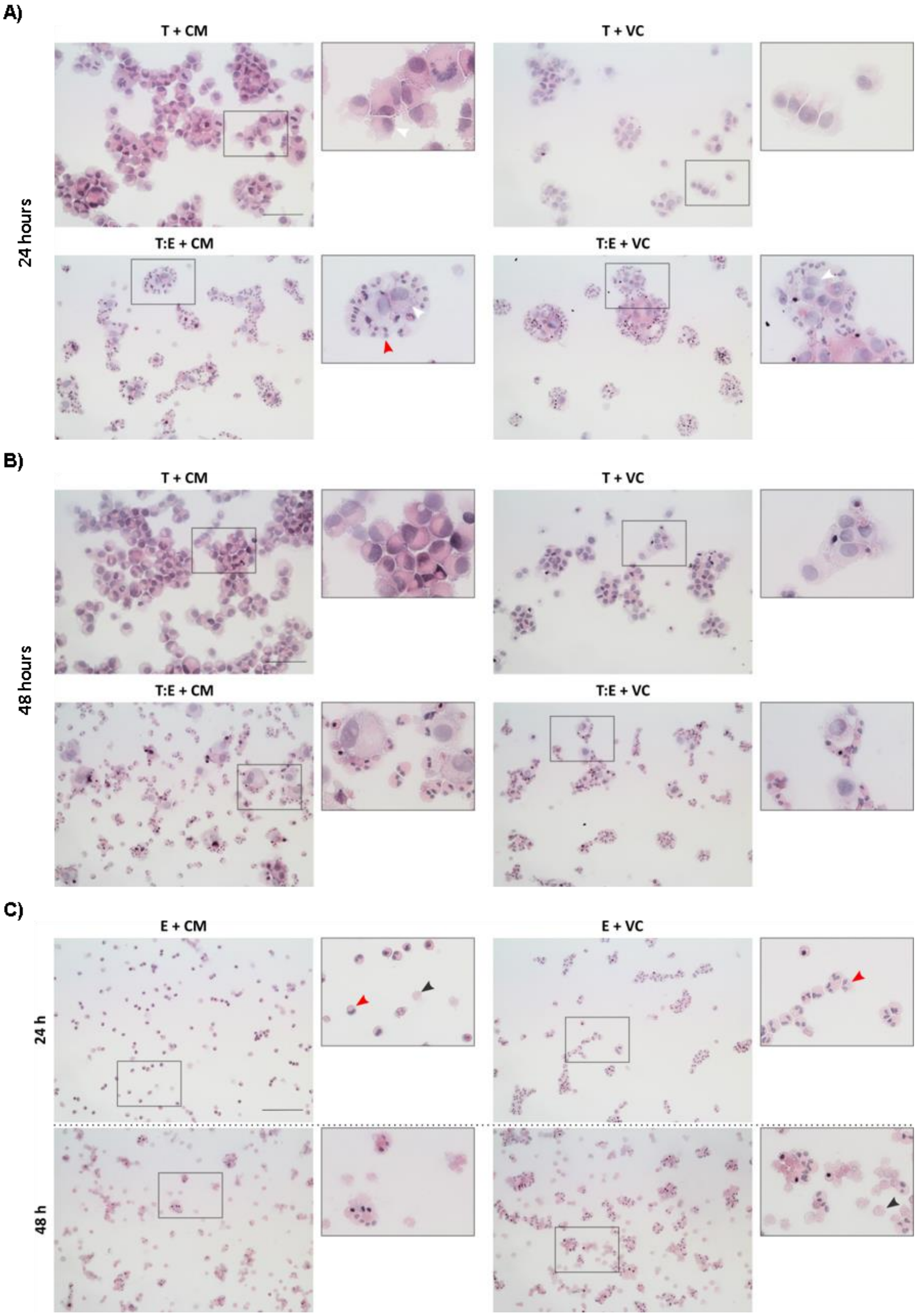

Figure S6. Eosinophils and melanoma cells form aggregates in co-cultures. Cytospin staining with HE for (**A**) 24 hours and (**B**) 48 hours non-adherent (co-)culture of MaMel63a (T) and eosinophils (E) in a 1:7.5 ratio in medium (CM) or medium containing 1 µM vemurafenib and 100 nM cobimetinib (VC). (**C**) Eosinophils alone only form aggregates when cultured in vemurafenib and cobimetinib containing medium after 24 and 48 hours and after 48 hours culture in medium. Black boxes in original image indicate the image section which was used for the magnification (3.2X) shown on

the right side of the original image. White arrow points at melanoma cell. Red arrow points at intact eosinophil. Black arrow point at dead eosinophil. Scale bar 100 μm.
